# Supplementary material for: Dengue Virus Capsid Protein Facilitates Genome Compaction and Packaging
Source: Int J Mol Sci. 2023 May 2;24(9):8158. doi: 10.3390/ijms24098158 (PMC10179140; doi:10.3390/ijms24098158)
Supplement: Supplementary file 1 [file ijms-24-08158-s001.zip › ijms-2291003-supplementary.pdf]

SUPPORTING INFORMATION FILE

MANUSCRIPT TITLE: Dengue Virus Capsid Protein Facilitates Genome Compaction and Packaging

MANUSCRIPT AUTHORS & AFFILIATIONS: Priscilla L. S. Boon [1,2,†], Ana S. Martins [3,†], Xin Ni Lim [4], Francisco J. Enguita [3], Nuno C. Santos [3], Peter J. Bond [1,2], Yue Wan [4], Ivo C. Martins [3,*] and Roland G. Huber [1,*]

1 Bioinformatics Institute (BII), Agency for Science, Technology and Research (A*STAR), Singapore 138671, Singapore

2 Department of Biological Sciences (DBS), National University of Singapore (NUS), 16 Science Drive 4, Singapore 117558, Singapore

3 Instituto de Medicina Molecular, Faculdade de Medicina, Universidade de Lisboa, Av. Prof. Egas Moniz, 1649-028 Lisbon, Portugal

4 Genome Institute of Singapore (GIS), Agency for Science, Technology and Research (A*STAR), Singapore 138672, Singapore

* Correspondence: ivomartins@fm.ul.pt (I.C.M.); rghuber@bii.a-star.edu.sg (R.G.H.)

† These authors contributed equally to this work.

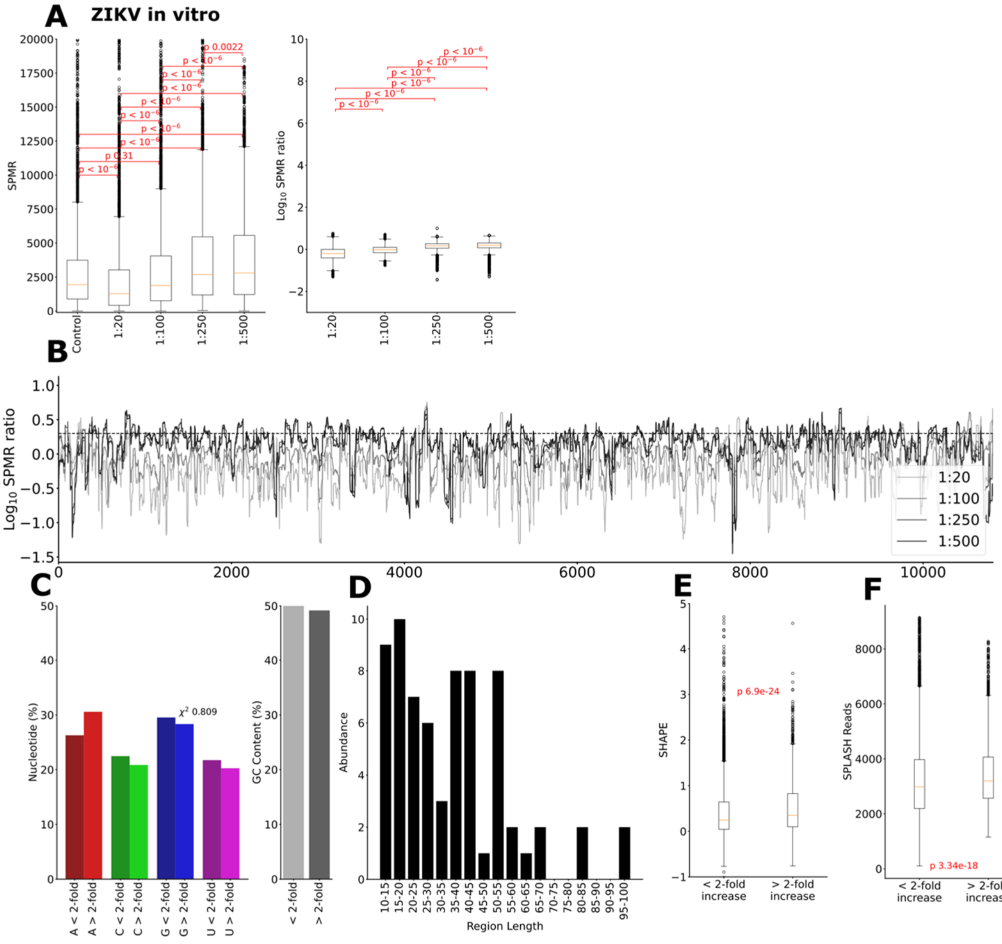

Figure S1. ZIKV C protein data. Abundance of (A) reads after SPMR normalization in ZIKV and ratio of read abundance over control show that at a molar ratio of 1:250 the genomes are saturated with C protein, indicating that the saturation point lies at or below this level. (B) Localization of binding signal along the ZIKV genome for *in vitro* transcribed genomic RNA. (C) The nucleotide composition and GC content of C protein binding locations is not significantly different from non-binding locations. (D) Average length of interacting segments shows multimodal distribution consistent with clusters of fixed-length interactions. (E) Binding *in vitro* preferentially occurs in higher SHAPE reactivity, single-stranded, and open regions of the viral genome. (F) ZIKV C shows *in vitro* preference for regions of long-range intramolecular RNA-RNA interactions, as measured by SPLASH.

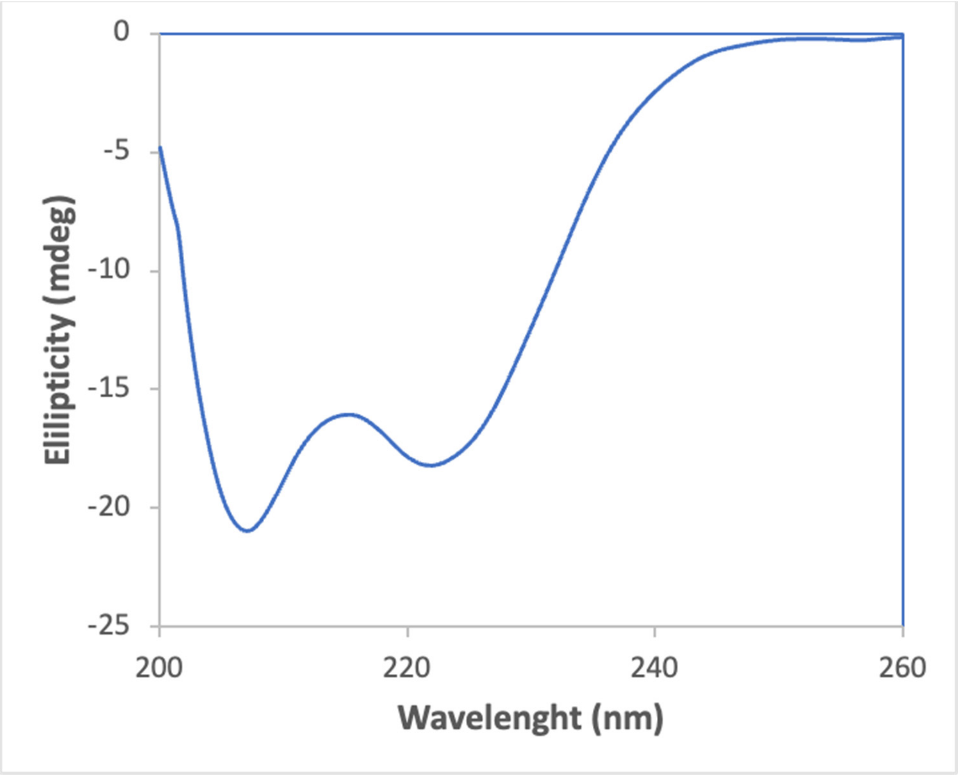

Figure S2. CD spectra obtained for ZIKV C protein at 25 °C. A typical spectrum for an α-helix secondary structure is obtained for ZIKV C protein at 25 °C (black line), as expected. CD measurements were performed as previously described for DENV C (Faustino et al. *Int J Mol Sci 20* (**2019**) 3870. doi:10.3390/ijms20163870), in 50 mM KH_2PO_4, 200 mM KCl, pH 7.5 buffer, at 25 °C.

Table S1. Overview of samples, conditions and number of aligned reads per sample.

**Experiment**	**Condition**	**Sample**	**Aligned Reads**
DENV FP	Control	RHH7758	1132561
DENV FP	Control	RHH7759	955739
DENV FP	1:20	RIS013	540638
DENV FP	1:20	RIS014	690978
DENV FP	1:100	RIS017	1655
DENV FP	1:100	RIS018	633412
DENV FP	1:250	RIS015	634442
DENV FP	1:250	RIS016	565531
DENV FP	1:500	RIS019	834199
DENV FP	1:500	RIS020	856445
ZIKV FP	Control	RHH5774	3286046
ZIKV FP	Control	RHH5775	1870611
ZIKV FP	1:20	RIS033	302268
ZIKV FP	1:20	RIS034	440390
ZIKV FP	1:100	RIS037	462708
ZIKV FP	1:100	RIS038	467441
ZIKV FP	1:250	RIS035	518893
ZIKV FP	1:250	RIS036	711872
ZIKV FP	1:500	RIS039	747313
ZIKV FP	1:500	RIS040	868036
DENV X-link	Background	RVH206	12623591
DENV X-link	Background	RVH207	6602451
DENV X-link	Control	RVH208	12132361
DENV X-link	Control	RVH209	8118898
DENV X-link	X-linked	RVH210	49213149
DENV X-link	X-linked	RVH211	70714181

Table S2. Correlation of SPMR between samples for all footprinting and crosslinking experiments.

**Experiment**	**Condition**	**Sample 1**	**Sample 2**	**Pearson r**
DENV FP	Control	RHH7758	RHH7759	0.999
DENV FP	1:20	RIS013	RIS014	0.833
DENV FP	1:100	RIS017	RIS018	0.953
DENV FP	1:250	RIS015	RIS016	0.866
DENV FP	1:500	RIS019	RIS020	0.974
ZIKV FP	Control	RHH5774	RHH5775	0.818
ZIKV FP	1:20	RIS033	RIS034	0.651
ZIKV FP	1:100	RIS037	RIS038	0.856
ZIKV FP	1:250	RIS035	RIS036	0.928
ZIKV FP	1:500	RIS039	RIS040	0.986
DENV X-link	Background	RVH206	RVH207	0.941
DENV X-link	Control	RVH208	RVH209	0.958
DENV X-link	X-linked	RVH210	RVH211	0.946
